# Surgical management of abdominal desmoids: a systematic review and meta-analysis

**DOI:** 10.1007/s11845-022-03008-8

**Published:** 2022-04-21

**Authors:** Dave Moore, Lucy Burns, Ben Creavin, Eanna Ryan, Kevin Conlon, Michael Eamon Kelly, Dara Kavanagh

**Affiliations:** 1 Department Surgery, Tallaght University Hospital, Tallaght, Dublin, D24 NR04 Ireland; 2 Royal College of Surgeons in Ireland, 123 St Stephens Green, Dublin 2, Ireland

**Keywords:** Desmoid tumours, Morbidity, Recurrence, Surgical management, Surgical outcomes

## Abstract

**Background:**

Desmoid tumours are benign fibromatous tumours arising from dysregulated myofibroblast proliferation within musculoaponeurotic structures. They can occur sporadically but more commonly are associated with genetic syndromes such as familial adenomatous polyposis (Sakorafas et al. in Surg Oncol 16(2):131–142, 2007) (FAP). Mutations in either the Wnt, β-catenin or *APC* genes are ‘key’ triggers for the development of these tumours (Howard and Pollock in Oncol Ther 4(1):57–72, 2016). Classically, these tumours do not metastasise; however, they are associated with significant morbidity and mortality due to their infiltrative pattern and/or local invasion. Historically, surgical resection was the cornerstone of treatment. There remains paucity of data regarding outcomes following the surgical management of abdominal desmoid tumours in terms of success, recurrence and morbidity.

**Objectives:**

The aim of this review was to assess the current evidence for surgical management of abdominal desmoid tumours in terms of success, recurrence and morbidity.

**Methods:**

A systematic search of articles in PubMed, EMBASE and The Cochrane Library databases was performed according to the Preferred Reporting Items for Systematic Reviews and Meta-Analyses guidelines for the period from January 2000 to November 2020.

**Results:**

Twenty-three studies were included, of which, 749 patients had surgical resection (696 for primary and 53 for recurrent desmoids), 243 patients (18.8%) were medically managed and 353 patients (27.3%) underwent surveillance. Median follow-up was 51.4 months (range 1–372). Six-hundred and ninety-six of the 749 resections (92.9%) underwent primary desmoid resection, with the remaining 53 (7.1%) undergoing resection for recurrence. One-hundred and two surgically managed patients (19%) developed a (re)recurrence, with mesenteric involvement the commonest site for recurrence (55%). When comparing recurrence post-surgery to progression following medical therapy, there was a trend towards better outcomes with surgery, with 25% of surgical patients having a recurrence versus 50.5% having progression with medical therapy [OR 0.40 (95% CI 0.06–2.70), *p* = 0.35]. Major morbidity following surgery was 4.4% (*n* = 33) with 2% (*n* = 14) mortality within 30 days of resection.

**Conclusion:**

The management of desmoids has considerable heterogeneity. Surgical resection for abdominal desmoids remains a valid treatment option in highly selective cases where negative margins can be obtained, with low major morbidity and/or mortality.

## Introduction

Desmoid tumours are a benign fibromatous tumour arising from dysregulated myofibroblast proliferation [1] within musculoaponeurotic structures. They are typically classified according to their location: extra-abdominal, abdominal or intra-abdominal. The latter is subclassified further into mesenteric fibromatosis and pelvic fibromatosis [2]. Classically, these tumours do not metastasise, but can cause significant morbidity and mortality due to their infiltrative pattern and local invasion into nearby structures.

Desmoid tumours are rare, with an annual incidence of 2–4 [3, 4] cases per million. They account for less than 3% of all soft tissue tumours [3], with 37–50% of them being intra-abdominal [4]. They can occur sporadically following trauma, abdominal surgery or post-partum, but more commonly are associated with genetic syndromes such as familial adenomatous polyposis [1] (FAP). Mutations in either the Wnt, β-catenin or *APC* genes are ‘key’ triggers in the dysregulated proliferation of fibroblasts and the development of these tumours [5]. There have been two separate entities described, sporadic with a CTNNB1 mutation v those with a germline APC mutation. Despite most being slow-growing, they can infiltrate surrounding organs/neurovascular structures, causing significant quality of life issues and morbidity or mortality.

Historically, surgical resection with the aim of achieving histologically negative margins has been the cornerstone of therapy. However, in recent years there has been a shift towards a conservative observational approach augmented with medical/chemotherapeutics in ‘select’ cases. Recurrence rates following resection are approximately 20% and ranges from 5 to 63% across the literature [9]. Systemic therapies such as hormonal agents, non-steroidal anti-inflammatory drugs or chemotherapy have been increasingly used along with selective use of radiotherapy. Several small studies have reported success with the use of NSAIDs such as sulindac [54]. Other studies have shown favourable clinical outcomes with high-dose SERMs compared to low dose but due to the paucity of supportive data there remains no consensus as to what the most effective therapeutic option is [43]. Sorafenib has been shown to prolong progression free survival in patients with symptomatic, progressive or refractory disease [56] whilst anthracycline chemotherapeutic regimens are associated with higher radiological response rate against desmoid tumours than other systemic agents [57]. A recent trend towards ‘watchful waiting’ is a reasonable management option in asymptomatic patients as spontaneous tumour regression may occur [1]. Complete excision is the treatment of choice for symptomatic tumours, although, this is associated with considerable morbidity. There remains a lack of evidence regarding post-operative recurrence [6–8].

The aim of this review was to assess the current evidence for surgical management of abdominal desmoid tumours in terms of success, recurrence and morbidity.

## Methods

**Fig. 1:**
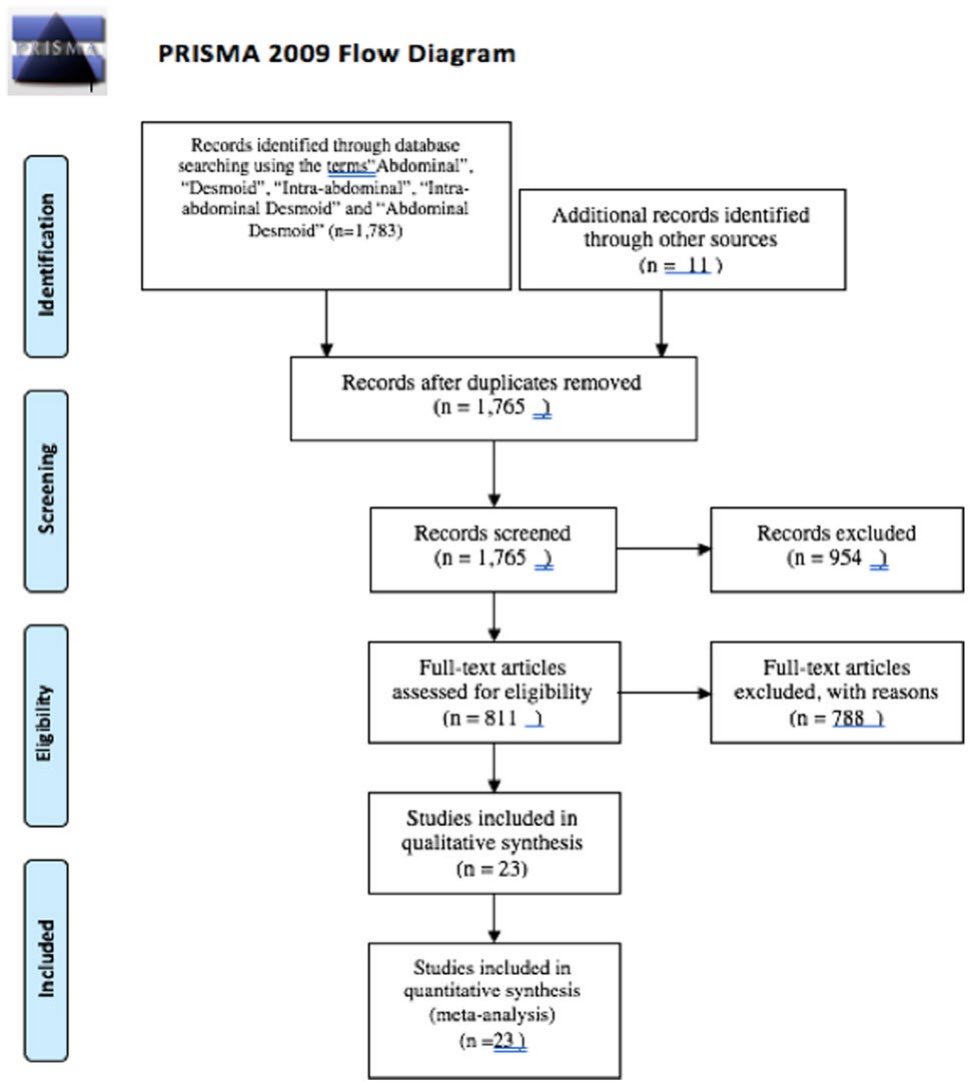
PRISMA flow diagram

A systematic review was performed according to the guidelines and recommendations from the Preferred Reporting Items for Systematic Reviews and Meta-Analyses checklist (PRISMA) [10] (see Fig. 1 and checklist shown in Supplementary Table 1). Institutional review board approval was not required.

### Search strategy

An electronic search for relevant publications was performed by two independent reviewers using the following resources: PubMed, Embase, Ovid and the Cochrane collaboration database from January 2000 to January 2020. The following search headings were used: ‘Abdominal’, ‘Desmoid’, ‘Intra-abdominal’, ‘Intra-abdominal Desmoid’ and ‘Abdominal Desmoid’. All titles were initially screened and appropriate abstracts were reviewed. Each of the relevant publication reference section and Google Scholar was also screened for other applicable publications. The last date of search was November 4th 2020.

### Inclusion criteria

To be included in the analysis, the studies had to meet the following criteria: (a) report on patients with either intra-abdominal, abdominal wall or pelvic desmoid fibromatosis; (b) all papers regarding the approaches for management of desmoid fibromatosis (upfront surgery v medical v observation); (c) patient populations across the studies must be matched and (d) report on recurrence rates post-surgical or medical therapy.

### Exclusion criteria

Studies were excluded from the analysis if: (a) they did not specify that these patients had intra-abdominal, abdominal wall or pelvic desmoids; (b) they did not report on recurrence rates post-treatment; (c) outcomes of interest were not reported; (d) the methodology was not clearly reported and (e) the data was overlapping among authors.

### Data extraction

Two reviewers (DM and BC) independently reviewed the literature according to the above predefined strategy and criteria. Each reviewer extracted the following data variables: title and reference details (first author, journal, year, country, PMID), study population characteristics (number in study, number treated by each approach, gender and age), disease characteristics and treatment specifics (number of desmoids treated surgically, number treated medically, type of surgical resection, those achieving an RO resection) and post-operative outcome data (morbidity, mortality, survival). All data was also recorded independently by both reviewers in separate databases and were compared at the end of the reviewing process to limit selection bias. The database was also reviewed by a third person (MK). Duplicates were removed and any disparities were clarified.

### Population examined

Patients with either intra-abdominal, abdominal wall or pelvic desmoid fibromatosis; defined as a benign fibromatous tumour arising from dysregulated myofibroblast proliferation [1] within musculoaponeurotic structures.

### Outcomes of interest

The following outcomes were used in the meta-analysis to ascertain the success of surgery in managing desmoid fibromatosis:

- Primary outcomes:
  1. Recurrence rates post-surgery (comparing primary resection v secondary resection)
- Secondary outcomes:
  2. Location of recurrence
  3. Negative margin (R0)
  4. Perioperative morbidity and/or mortality (Clavien-Dindo classification)
  5. Tumour regression with medical therapy

### Statistical analysis

Statistical analysis was performed using Revman Statistical Software (Ver. 5 Copenhagen, Denmark). Binary outcome data were reported as odd ratios (OR) and 95% confidence interval (95% CI) was estimated using the Mantel–Haenszel method. For continuous data, mean differences and 95% CI were estimated using inverse variance weighting. Outcome measures (mean + standard deviation and median + interquartile range) were recorded. If needed, outcome variables (mean and SD) were estimated from the median and range using formula described by Gronchi et al. [19]. Heterogeneity was assessed by *I*-squared statistics, with > 50% being considered as considerable heterogeneity. Statistical significance was attributed to *p* value < 0.05. The quality of the studies included in this systematic review was assessed using the methodological index for non-randomized studies (MINORS) score [27]. The quality score rating was determined for each publication and recorded. We also calculated recurrence based on weights, with each study being allocated a weight based on sample size.

## Results

### Eligible studies

**Fig. 2:**
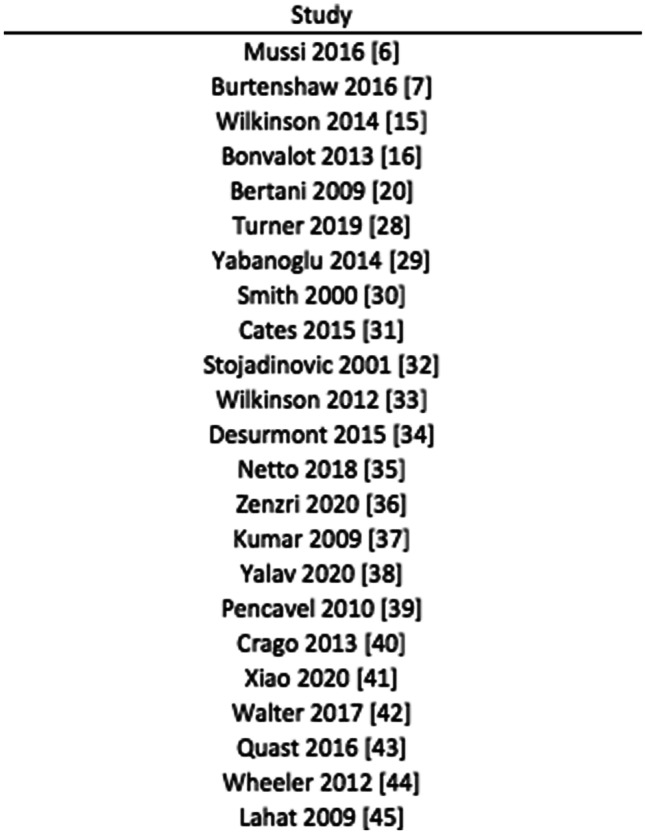
List of studies included for analysis

A total of 1,783 articles were initially identified using the aforementioned search strategy (Fig. 1). On full text screening, 23 publications (see Fig. 2) that met the inclusion criteria were included in the meta-analysis. On review of the extracted data, there was 100% agreement between the reviewers.

### Demographics

Analysis was performed on 1,292 patients. Seven-hundred and forty-nine intra-abdominal desmoid tumour resections were carried out of which 142 patients (19%) developed a (re)recurrence following surgery for primary or recurrent desmoid disease. A total 696 (53.8%) had upfront surgical resection, 243 (18.8%) were treated medically and 353 (27.3%) underwent surveillance (watchful waiting). The studies spanned a mean time period of 19.6 years (see Table 1 for study characteristics). Across all studies, female gender was more common, accounting for 71% (*n* = 907) of all patients. The median age of patients with desmoid tumours was 35.3 years old (mean age 37.9).

**Table 1:** Study characteristics

	**Study**	**Numbers treated surgically/medically**	**Year**	**Study period**	**Gender (M/F)**	**Mean age ± S.D [median/IQR]**
1	Xiao et al. [41]	(12/3)	2020	Mar 1983–Sep 2018	(8/8)	39/N.S
2	Yalav et al. [38]	(11/0)	2020	2010–2019	(5/6)	44.2 ± 15.8
3	Zenzri et al. [36]	(30/0)	2020	Feb 2000–Nov 2019	(2/28)	[35/18–80]
4	Turner et al. [28]	(25/0)	2019	Aug 2004–Sep 2015	(14/39)	42/N.S
5	Netto et al. [35]	(28/5)	2018	1982–2014	(3/24)	34/19–88
6	Walter et al. [42]	(36/13)	2017	1965–2013	(11/20)	[25/13–76]
7	Burtenshaw et al. [7]	(70/0)	2016	1980–2012	(20/47)	[36/14–83]
8	Mussi et al. [6]	(30/0)	2016	Jan 2000–Sep 2013	(5/28)	[37/28–61]
9	Quast et al. [43]	(0/134)	2016	2003–2015	44/90	35.54 ± 13.7
10	Cates [31]	(29/4)	2015	1983–2011	(13/16)	33.8 ± 24.3
11	Desurmont et al. [34]	(17/80)	2015	1970–2010	(34/45)	33.4 ± 13.05
12	Wilkinson et al. [15]	(50/0)	2014	1998–2013	(2/48)	[36/15–64]
13	Yabanoglu et al. [29]	(13/0)	2014	1997–2013	(2/11)	36 ± 14
14	Bonvalot et al. [16]	(43/4)	2013	1993–2012	(1/40)	[34/18–74]
15	Crago et al. [40]	(171/0)	2013	1982–2011	(166/329)	N.S
16	Wheeler et al. [44]	(18/0)	2012	Not specified	N.S	N.S
17	Wilkinson et al. [33]	(15/0)	2012	2001–2011	(6/9)	[42/19–64]
18	Pencavel et al. [39]	(30/0)	2010	2000–2009	(2/28)	[35]
19	Bertani et al. [20]	(14/0)	2009	Oct 1999–Jun 2008	(3/11)	41.5/16–57
20	Kumar et al. [37]	(30/0)	2009	1995–2004	(10/22)	N.S
21	Lahat et al. [45]	(16/0)	2009	1995–2009	(4/12)	40.5/24–70
22	Smith et al. [30]	(20/0)	2000	Jul 1982–Jun 1998	(12/12)	[39/18–73]
23	Stojadinovic et al. [32]	(41/0)	2001	Jul 1982–Aug 1999	(5/34)	[34/23–79]
				Range 1970–2019	(372/907)	37.9 [35.3]

*N.S* not specified

### Primary surgery group

Of the 696 patients (92.9% of the study cohort) undergoing primary resection, there was a recurrence rate of 17.7% (*n* = 123) when weighted appropriately for study numbers the recurrence rate remained at 17.7% for upfront surgery. Expectantly, the majority of recurrences occurred intra-abdominally, with most involving the mesentery (55.3%), and/or the abdominal wall in one third of cases.

Nineteen studies reported on the negative (R_0_) margin rates following surgical resection. R_0_ rates were reported in 44.4% of the primary resection group. Follow-up was reported in twenty-two studies, with a median follow-up of 50.7 months in the primary surgery group.

Many studies report on the clinical presentation of the tumour, such as a palpable mass, abdominal pain and weight loss [32, 33, 38, 45].

However, specific indications for surgery were not recorded in the majority of studies where patients were operated on with curative intent [15, 32, 33, 45].

Some studies reported on why patients were not managed surgically or where treatment was changed to surgical management, e.g. proximity to vasculature, tumour size, patient preference or degree of symptoms [6, 16, 41]. In terms of emergency surgery, only three studies commented on this with 3 patients having an acute abdomen [33, 38] and 11 operated on in an emergency setting or with palliative intent [35].

### Recurrence surgery group

Fifty-three (7.1%) patients underwent surgery for a recurrence following index surgery. Thirty-four percent (*n* = 18) of the fifty-three having surgery for recurrent desmoid had a re-recurrence as shown in Table 2. When weighted appropriately for study numbers, the recurrence rate was 33.9% for the re-recurrence group. Of note only 5 of the re-recurrence papers reported on resection margins with only one of the papers reporting an R0 resection. As mentioned previously, follow-up was reported in twenty-two studies and the median follow-up was 52 months in the recurrence surgery group. R_0_ rates were reported in only 5.7% in the recurrence group. Table 3 shows the recurrences post-surgery with Table 4 the weighted recurrences.

**Table 2:** Recurrence surgery group

**Paper**	***N*** **= surgery**	**Recurrence (%)**	**Site of recurrence** **(Abdo-wall/intra-abdominal)**	**R0 [R1/R2]**	**Post-op. morbidity**	**Post-op. mortality**	**Mean follow-up [median/IQR]**
**Mussi et al. **[6]	30	3 (10%)	[3/0]	24 [3]	3	0	42/1–49
**Burtenshaw et al. **[7]	67	16 (23.8%)	[2/14]	14 [2]	N.S	N.S	[22/8–220]
**Turner et al. **[28]	25	1 (4%)	[0/1]	5 [8]	N.S	N.S	[35/1–137]
**Yabanoglu et al. **[29]	13	3 (23.1%)	[3/0]	11 [2]	0	0	56.7 [3–177]
**Smith et al. **[30]	16	6 (37.5%)	[0/6]	3 [11]	8	1	[62/N.S]
**Bonvalot et al. **[16]	41	1 (2.4%)	[1/0]	23 [0]	0	0	[97/9–226]
**Wilkinson et al. **[15]	50	4 (8%)	[4/0]	22 [28]	2	0	[6/12–180]
**Stojadinovic et al. **[32]	39	3 (7.6%)	[3/0]	28 [11]	4	0	[53/4–212]
**Wilkinson et al. **[33]	15	2 (13.3%)	[2/0]	2 [13]	1	0	[40/6–119]
**Bertani et al. **[20]	14	0 (0%)	[0/0]	13 [0]	0	0	48.8 [55/11–108]
**Desurmont et al. **[34]	17	3 (17.6%)	[0/3]	6 (N.S)	0	2	89.4 ± 76.2 [72.6/1.7–280]
**Netto et al. **[35]	27	3 (11.1%)	[3/0]	21 [6]	8	0	82 [N.S]
**Zenzri et al. **[36]	27	11 (40.7%)	N.S	14 [13]	N.S	N.S	[21/1–60]
**Kumar et al. **[37]	22	8 (36.3%)	[8/0]	16 [6]	N.S	N.S	N.S/range 12–120
**Yalav et al. **[38]	11	1 (9.1%)	[0/1]	10 [1]	0	1	43.4 ± 28.4
**Pencavel et al. **[39]	30	0 (0%)	[0/0]	15 [15]	1	0	[39.2/7–107]
**Crago et al. **[40]	171	31 (18.1%)	[8/23]	57 [43]	N.S	6	[60/0–327]
**Xiao et al. **[41]	11	1 (9.1%)	[0/1]	10 [1]	N.S	N.S	23/range 4–72
**Walter et al. **[42]	36	26 (72.2%)	[0/20]	N.S	N.S	N.S	[108/1.2–372]
**Wheeler et al. **[44]	18	0 (0%)	[0/0]	N.S	1	4	[27/1–161]
**Lahat et al. **[45]	16	0 (0%)	[0/0]	15 [1]	5	0	[64/5–143]
	696	123 (17.7%)	(37/68)	R0 = 309	33 (4.7%)	14 (2%)	55.04 [50.7/1–372]

**Recurrence post-secondary resection**							
**Paper**	***N* = surgery**	**Recurrence (%)**	**Site of recurrence (A/B)**	**R0 [R1]**	**Post-op morb**	**Post-op mort**	**Mean follow-up [median/IQR]**
**Cates **[31]	29	9 (31.1%)	N.S	3 [5]	N.S	N.S	[43.2/2.5–267.6]
**Smith et al. **[30]	4	1 (25%)	[0/1]	N.S	0	1	[62/N.S]
**Burtenshaw et al. **[7]	3	0 (0%)	N.S	[9]	N.S	N.S	[17/8–19]
**Bonvalot et al. **[16]	2	0 (0%)	N.S	N.S	N.S	0	[97/9–226]
**Kumar et al. **[37]	8	3 (37.5%)	N.S	N.S	N.S	N.S	N.S [12–120]
**Zenzri et al. **[36]	3	1 (33.3%)	N.S	0 [3]	N.S	N.S	[21/1–60]
**Stojadinovic et al. **[32]	2	2 (100%)	[2/0]	0 [2]	N.S	0	[53/4–212]
**Netto et al. **[35]	1	1 (100%)	[1/0]	0 [1]	N.S	N.S	82 [N.S]
**Xiao et al. **[41]	1	1 (100%)	[0/1]	N.S	N.S	N.S	23/[4–72]
	53	18 (34%)	[3/2]	R0 = 3	0	1 (1.9%)	52.5 [48.9/1–267.6]

**Table 3:** Weighted recurrences post-primary resection

**Paper**	***N*** **= surgery**	**Recurrence post-surgery**	**Weight %**	**Recurrence (%)**
**Mussi et al. **[6]	30	3	4.3	10
**Burtenshaw et al. **[7]	67	16	9.5	23.8
**Turner et al. **[28]	25	1	3.6	4
**Yabanoglu et al. **[29]	13	3	1.9	23
**Smith et al. **[30]	16	6	2.3	37.5
**Bonvalot et al. **[16]	41	1	5.9	2.4
**Wilkinson et al. **[15]	50	4	7.2	8
**Stojadinovic et al. **[32]	39	3	5.6	7.7
**Wilkinson et al. **[33]	15	2	2.2	13.3
**Bertani et al. **[20]	14	0	2	0
**Desurmont et al. **[34]	17	3	2.4	17.6
**Netto et al. **[35]	27	3	3.9	11.1
**Zenzri et al. **[36]	27	11	3.9	40.7
**Kumar et al. **[37]	22	8	3.2	36.4
**Yalav et al. **[38]	11	1	1.6	9
**Pencavel et al. **[39]	30	0	4.3	0
**Crago et al. **[40]	171	31	24.5	18.1
**Xiao et al. **[41]	11	1	1.6	9
**Walter et al. **[42]	36	26	5.2	72.2
**Wheeler et al. **[44]	18	0	2.6	0
**Lahat et al. **[45]	16	0	2.3	0
	696	123	100	Weighted^53^ = 17.7

**Weighted recurrence post-secondary resection**				
**Paper**	***N* = surgery**	**Recurrence post-surgery**	**Weight %**	**Recurrence (%)**
**Cates **[31]	29	9	54.6	31
**Smith et al. **[30]	4	1	7.6	25
**Burtenshaw et al. **[7]	3	0	5.7	0
**Bonvalot et al. **[16]	2	0	3.8	0
**Kumar et al. **[37]	8	3	15	37.5
**Zenzri et al. **[36]	3	1	5.7	33.3
**Stojadinovic et al. **[32]	2	2	3.8	100
**Netto et al. **[35]	1	1	1.9	100
**Xiao et al. **[41]	1	1	1.9	100
	53	18	100	Weighted^53^ = 33.9

**Table 4:** Medical outcomes

**Paper**	***N*= medical**	**Initial clinical response**	**Regrowth post-initial response**	**Morbidity [mortality]**
Bonvalot et al. [16]	4	0	0	N.S [0]
Cates [31]	4	2 (50%)	0	N.S [0]
Desurmont et al. [34]	80	34 (42.5%)	0	N.S
Netto et al. [35]	5	4 (80%)	4	0 [N.S]
Xiao et al. [41]	3	3 (100%)	0	0 [0]
Walter et al. [42]	13	11 (84.7%)	0	N.S
Quast et al. [43]	134	114 (85%)	0	16 [2]
	243	168 (69.1%)	4	16 [2]

### Medical outcomes

Across the included studies, 243 patients underwent medical treatment as their primary management. There was considerable heterogeneity in the choice of therapy used, including differing chemotherapeutic regimens, selective oestrogen receptor modulators and non-steroidal anti-inflammatory agents (Table 5).

**Table 5:** MINORS score

	Clearly stated aim	Inclusion of consecutive patients	Prospective data collection	Endpoints appropriate to study aim	Unbiased assessment of study endpoint	Follow-up period appropriate to study aim	< 5% lost to follow-up	Prospective calculation of study size	Adequate control group	Contemporary groups	Baseline equivalence of groups	Adequate statistical analyses	Total
Mussi et al. [6]	2	2	1	2	1	1	2	2	N/A	N/A	N/A	N/A	13
Burtenshaw et al. [7]	2	2	1	2	1	1	2	2	1	2	1	0	17
Wilkinson et al. [33]	2	2	2	2	1	1	2	0	N/A	N/A	N/A	N/A	12
Bonvalot et al. [16]	2	1	0	2	1	1	2	2	1	2	2	2	18
Bertani et al. [20]	2	2	1	2	1	1	2	0	N/A	N/A	N/A	N/A	11
Turner et al.[8]	2	2	1	2	1	1	2	2	1	2	1	2	19
Yabanoglu et al. [29]	2	1	1	2	1	1	2	2	N/A	N/A	N/A	N/A	12
Smith et al. [30]	2	2	2	1	1	2	2	0	N/A	N/A	N/A	N/A	12
Cates [31]	2	2	1	2	1	1	2	2	N/A	N/A	N/A	N/A	13
Stojadinovic 2001	2	1	2	2	1	1	2	1	N/A	N/A	N/A	N/A	12
Wilkinson 2014	2	2	2	2	1	2	2	2	N/A	N/A	N/A	N/A	15
Desurmont 2015	1	2	1	1	1	2	2	2	1	2	1	0	16
Netto 2018	1	2	1	1	1	2	2	1	2	1	1	2	17
Zenzri 2020	2	2	1	2	1	1	1	2	N/A	N/A	N/A	N/A	12
Kumar 2009	2	2	1	1	1	1	2	0	N/A	N/A	N/A	N/A	10
Yalav 2020	2	1	1	1	1	1	2	2	N/A	N/A	N/A	N/A	11
Pencavel 2010	1	2	2	2	1	1	2	1	N/A	N/A	N/A	N/A	12
Crago 2013	2	2	2	2	1	2	2	2	2	1	1	1	20
Xiao 2020	2	2	1	2	1	1	1	0	N/A	N/A	N/A	N/A	10
Walter 2017	2	2	1	2	1	2	2	2	N/A	N/A	N/A	N/A	14
Quast 2016	2	2	2	2	1	2	2	2	N/A	N/A	N/A	N/A	15
Wheeler 2012	2	2	1	1	1	1	2	0	N/A	N/A	N/A	N/A	10
Lahat 2009	1	2	1	2	1	2	2	0	N/A	N/A	N/A	N/A	11

The items are scored 0 (not reported), 1 (reported but inadequate) or 2 (reported and adequate). The global ideal score being 16 for non-comparative studies and 24 for comparative studies [27]

Across the sixteen studies, 155 patients (63.8%) showed some tumour regression with medical therapy, with 75 patients (30.9%) having no response or a regrowth over the follow-up period. The morbidity rate associated with medical therapy was 6.6% (*n* = 16), with two patients (0.8%) dying as a result of complications of their treatment.

### Surgery v medical therapy

**Fig. 3:**
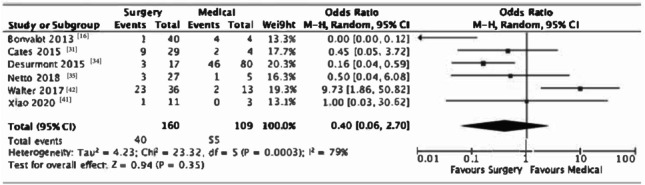
List of studies comparing surgery with medical treatment

Six studies directly compared recurrence rates post-resection to disease progression following medical therapy. Overall, there was a trend towards better outcomes with surgery, with 25% of surgical patients having a recurrence (*n* = 40/160) versus 50.5% (*n* = 55/109) having progression of disease with medical therapy [OR 0.40 (95% CI 0.06–2.70), *p* = 0.35]. As expected there was considerable heterogeneity across the studies (*I*^2^ = 79%, *p* = 0.0003). Figure 3 shows a list of studies comparing surgery with medical treatment.

## Discussion

Recurrence rates following resection are previously reported as 20% ranging from 5 to 63% across the literature [9]. Systemic therapies such as hormonal agents, non-steroidal anti-inflammatory drugs or chemotherapy have been increasingly used along with selective use of radiotherapy. Our review looked at 749 intra-abdominal desmoid tumour resections over a 19-year period out of which 142 patients (19%) developed a (re)recurrence following surgery for primary or recurrent desmoid disease. We showed a 17.7% recurrence in the primary surgical group with the majority of recurrences occurring intra-abdominally, compared with 34% in those undergoing surgery for a recurrent desmoid tumour. With the large range of medical therapies which we did not concentrate on in this review, 155 patients (63.8%) showed some tumour regression, with 75 patients (30.9%) having no response or a regrowth over the follow-up period. In those studies that compared recurrence rates post-resection to disease progression following medical therapy, there was a trend towards better outcomes with surgery, with 25% of surgical patients having a recurrence (*n* = 40/160) versus 50.5% (*n* = 55/109) having progression of disease with medical therapy. These findings support role of surgical resection remains a valid treatment option when negative margins can be obtained.

The management of desmoid tumours across the literature is heterogenous, lacking any international clarity and consensus on definitively when to opt for surgery over medical therapy and vice versa [11, 14, 15, 30, 54]. As evident in the current study, the majority of data comes from small volume, single-centre series, grouping differing desmoid (anatomical) locations together, along with reporting on various medical therapies. Furthermore, the median follow-up and method of surveillance significantly differ across the studies included.

Historically, surgery has been the cornerstone of treatment, but concerns over resectability and recurrence are appreciable. This review has demonstrated recurrence rates of 17% and 34% in those having surgery for the management of primary and recurrent desmoid tumours, respectively. This is largely attributable to either biological behaviour or due to the negative surgical margins. As a result, alternative management strategies and medical therapies have been explored [11, 15–17, 19, 23, 43]. The decision-making regarding ‘best’ management remains challenging, nuanced and should balance the potential morbidity that a treatment could cause against the infiltrative nature of the desmoid tumour [14].

It is difficult to determine which patients would derive the most benefit from surgical management, given the rarity of the disease, the variability of tumour behaviour from aggressive disease to spontaneous regression, as well as different anatomical site and size of the tumour, thus it necessitates individualized treatment options [6, 7, 15, 16, 19].

Furthermore, given that the cohort is largely female and of reproductive age, the associated morbidity associated with medical and surgical treatment options must be considered [7, 15]. We must also acknowledge the historical bias towards surgical management represented in these studies given that traditionally it has been the primary treatment for patients with resectable desmoid tumours [7, 16, 35].

Intra-abdominal, abdominal wall and pelvic desmoid tumours pose a particularly difficult challenge when compared with peripheral desmoids [16, 23]. Whilst the aim is to cure in order to prevent, enhance progression free and overall survival and also to alleviate associated symptoms, their proximity to vascular and/or gastrointestinal structures can limit resection margins. Ultimately, decision-making regarding optimal management should balance morbidity of therapy versus morbidity with progression and symptomatology at presentation. The involvement of a specialist multidisciplinary team including comprehensive diagnostics are ‘key’ factors to ensure correct management approach [46]. Factors like extensive (central) mesenteric involvement or involvement of neurovascular structures should be highlighted and therefore ensure operative management is not pursued, unless there are significant indications [14]. Historically, there have been incidences where extensive small bowel resection has resulted in short gut syndrome, requiring subsequent small intestinal transplantation [14]. Other factors associated with higher recurrence include size (> 7 cm) [15–18] invasion of major vessels and/or nerves and involvement of the surgical margins [47].

Another issue to consider when contemplating the appropriate management approach is the expected natural evolution of desmoid tumours [7, 9, 16, 17]. Some desmoids have a more stable, indolent growth phase, others more aggressive and some have spontaneous regression [16, 23]. To date, there is no accurate biomarker to predict involution, progression or response to therapeutics [15]. *CTNNB1* is the gene which encodes for beta-catenin [6]. Beta-catenin mutations have been suggested to have poorer prognosis but further research is needed to validate this theory [15]. Surgical resection was once considered the mainstay of therapy for desmoid fibromatosis, but within the last decade a shift towards active surveillance has been promoted as this subtype tend to have a prolonged stable disease phase, with some having spontaneous regression when left alone [15]. As a result, most would advocate a phase of active surveillance initially in all desmoids, unless there are extenuating circumstances [7–9, 16–19, 23]. Interval 4–6 monthly clinical review supported with cross-sectional imaging (magnetic resonance imaging and/or computed tomography), help document growth, stability or regressions [15].

Interestingly, Bonvalot et al. found one third of the patients who were managed with active surveillance remained stable, and one third were found to have spontaneous regression, this presents a good argument for a period of active surveillance to allow for appropriate selection of patients who would best benefit from surgical management, by adapting treatment based on tumour behaviour and patient’s symptoms [16, 19].

The limitation to active surveillance is determining which patients have aggressive disease and when is the appropriate time to shift treatment strategy, with the risk that some patients could be hindered by delayed treatment [6, 16]. They also found that initial tumour size was related to an early change in treatment strategy away from surveillance [16].

Furthermore, the French Sarcoma Group reported the same finding and advised for treatment from presentation for tumours > 7 cm. The algorithm from the French (FSG) and Italian (ISG) sarcoma groups suggested treatment strategy according to the site of the tumour as different anatomical locations have different associated risks of recurrence and morbidity [6, 7, 15, 19].

Whilst we have shown that medical therapy can achieve some stability or regression in approximately 60% of cases, some of these treatments are not without complication [43]. Complications following chemotherapy (fertility issues, cardiotoxicity with prolonged use and nausea and vomiting) and hormonal treatments (fertility issues, venous thromboembolism) are appreciable [48–51]. It is notable that the majority of females across the included studies are within child-bearing age and therefore the use of some of these therapies would have considerable impact to quality of life [43, 48–51]. For this reason, surgery in ‘select’ cases still has a role such as for patients with swift progression, important organ involvement, severe complications and symptoms [16, 20, 29, 32, 39, 41, 44, 45]. In addition, multiple studies have demonstrated successful pregnancies following surgery for desmoid fibromatosis [15, 24–26]. Overall, the choice (if any at all) of treatment and the timing must be decided on a case-by-case basis, with good patient education and counselling [32–38, 46].

The role of surgery in the management of recurrent desmoids is controversial. Prior resection, fibrosis and pattern of recurrence can make further surgery more difficult [15–20, 26]. Across the included studies, only 6% of patients having re-do surgery had a R_0_ resection. A total of 34% developed a re-recurrence following surgery for recurrent desmoid tumours despite 94% R_1_ rates perhaps this could be explained by the biologically heterogeneity of the group hence why there is a more nuanced approach in managing these. Unfortunately, it is not clear from the literature the indication for re-do surgery, or the multidisciplinary decision-making process in these cases. In this review, only 5 of the re-recurrence papers reported on resection margins with only one of the papers reporting an R_0_ resection suggesting re-resection is not a good option. Despite this, some patients with recurrent disease still live for extended periods which is why surgery still is part of the armamentarium of treatment options [15, 18, 20, 28].

There are conflicting reports across studies regarding the relationship of negative margins, to disease recurrence, several studies results supported the idea that surgical margin status is associated with recurrence or poor prognosis [8, 20, 32, 36, 41], whilst several studies did not [7, 15, 20, 31, 39]. Surgical management which maintains organ function, yet provides the maximal survival benefit may be more advantageous than R0 resection [8, 41].

Other factors predictive of tumour recurrence such as size and location must also be considered [7, 37, 40].

In relation, to the use of adjuvant radiotherapy in the setting of positive margins, it should be noted that surgical resection can require reconstruction using mesh [6, 15, 16, 32, 39], this would be a relative contraindication to irradiate mesh and skin, given the risk to the digestive system at this location [16]. Additionally, there are notable complications associated with radiotherapy such as wound complications, radiation enteritis and fibrosis, in an again a predominantly young patient cohort [34, 40]. However, some studies used adjuvant radiotherapy in the setting of positive margins, as they felt it was worth offsetting the potential risk associated with the margin status [36–38]. Furthermore, the lack of randomized data makes it difficult to assess the value of adjuvant treatment [37, 40].

Finally, the impact of surgery on quality of life is under-evaluated and prospective studies/registries are needed to clarify the validity of this strategy [52].

Bertani et al. was the only study to formally assess quality of life, using the European Organization for the Research and Treatment of Cancer (EORTC) QLQ-C30 questionnaire [20]. However, other studies discuss the early and late morbidity, associated with surgery such as hernia formation, pain secondary to mesh, bulging of mesh, need for reoperation, short gut syndrome and issues associated with pregnancy and delivery [6, 14–16, 24–26].

Ultimately the focus should be on event free survival and long-term disease control. A joint-global consensus was set out in 2020 stating that the primary approach to desmoids should be active surveillance for both sporadic and familial desmoids with surgery being the second line treatment but only for sporadic tumours of the abdominal wall [46].

We acknowledge that limitations to this review include that the management of desmoid tumours across the literature is heterogenous, with the majority of data coming from small volume, single-centre series, grouping differing desmoid (anatomical) locations together, along with reporting on various medical therapies. Furthermore, the median follow-up and method of surveillance significantly differ across the studies included. We must also acknowledge the historical bias towards surgical management represented in these studies with weighting not fully able make up for this bias [7, 16, 35].

## Conclusion

The management of desmoids has considerable heterogeneity in terms of surgical and medical strategies. In highly selective cases, the role of surgical resection remains a valid treatment option when negative margins can be obtained following a failed period of observation. Overall, it is associated with low major morbidity and/or mortality. The role of surgery in symptom palliation remains unclear.

## Data Availability

All data generated or analysed during this study are included in this article, the reference section and its supplementary material files. Further enquiries can be directed to the corresponding author.
