# Correction to: Surgical management of abdominal desmoids: a systematic review and meta‑analysis

**DOI:** 10.1007/s11845-022-03025-7

**Published:** 2022-05-13

**Authors:** Dave Moore, Lucy Burns, Ben Creavin, Eanna Ryan, Kevin Conlon, Michael Eamon Kelly, Dara Oliver Kavanagh

**Affiliations:** 1 Department Surgery, Tallaght University Hospital, Dublin, D24 NR04 Tallaght Ireland; 2 Royal College of Surgeons in Ireland, 123 St Stephens Green, Dublin 2, Ireland

## Correction to: Irish Journal of Medical Science (2022) https://doi.org/10.1007/s11845-022-03008-8

The online version contains an error. In the author group, the order of authors was incorrect. Mr Dara Kavanagh is the lead author and therefore should be named as the last author. This has now been corrected for all fields.

The original article has been corrected.

